# How to combine the use of intraoperative magnetic resonance imaging (MRI) and awake craniotomy for microsurgical resection of hemorrhagic cavernous malformation in eloquent area: a case report

**DOI:** 10.1186/s13256-023-03816-1

**Published:** 2023-04-12

**Authors:** Constantin Tuleasca, Iulia Peciu-Florianu, Ondine Strachowski, Benoit Derre, Quentin Vannod-Michel, Nicolas Reyns

**Affiliations:** 1 Neurosurgery and Neurooncology Service Lille, Centre Hospitalier Regional Universitaire de Lille, Roger Salengro Hospital, Lille, France; 2 Faculty of Biology and Medicine (FBM), University of Lausanne (UNIL), Lausanne, Switzerland; 3 Department of Clinical Neurosciences, Neurosurgery Service and Gamma Knife Center, Lausanne University Hospital (CHUV), Lausanne, Switzerland; 4 Signal Processing Laboratory (LTS 5), Ecole Polytechnique Fédérale de Lausanne (EPFL), Lausanne, Switzerland; 5 Neuroradiology Service, Centre Hospitalier Regional Universitaire de Lille, Roger Salengro Hospital, Lille, France

**Keywords:** Cavernoma, Hemorrhage, Awake, Intraoperative MRI, Surgery

## Abstract

**Background:**

Cavernous malformations are clusters of abnormal and hyalinized capillaries without interfering brain tissue. Here, we present a cavernous malformation operated under awake conditions, due to location, in an eloquent area and using intraoperative magnetic resonance imaging due to patient’s movement upon the awake phase.

**Case presentation:**

We present the pre-, per-, and postoperative course of an inferior parietal cavernous malformation, located in eloquent area, in a 27-year-old right-handed Caucasian male, presenting with intralesional hemorrhage and epilepsy. Preoperative diffusion tensor imaging has shown the cavernous malformation at the interface between the arcuate fasciculus and the inferior fronto-occipital fasciculus. We describe the microsurgical approach, combining preoperative diffusion tensor imaging, neuronavigation, awake microsurgical resection, and intraoperative magnetic resonance imaging.

**Conclusion:**

Complete microsurgical en bloc resection has been performed and is feasible even in eloquent locations. Intraoperative magnetic resonance imaging was considered an important adjunct, particularly used in this case as the patient moved during the “awake" phase of the surgery and thus neuronavigation was not accurate anymore. Postoperative course was marked by a unique, generalized seizure without any adverse event. Immediate and 3 months postoperative magnetic resonance imaging confirmed the absence of any residue. Pre- and postoperative neuropsychological exams were unremarkable.

**Supplementary Information:**

The online version contains supplementary material available at 10.1186/s13256-023-03816-1.

## Background

Cerebral cavernous malformations (CCMs) assemble abnormal and hyalinized capillaries, without interfering brain tissue. Hemosiderin deposits and gliosis classically surround them, due to intermittent microhemorrhages and thrombosis [6]. CCMs are found incidentally, but can also cause symptomatic hemorrhage or seizure as the most classic clinical manifestations [6].

The average annual rate of hemorrhage is 0.7–1.1% per lesion in patients without prior bleeding history. This risk increases to 4.5% if sustained previous intracerebral hemorrhage was present [5].

Microsurgical resection of CCM located in eloquent areas may be dangerous [20], due to brain tissue shift during the intervention, the use of brain retractors, but also other aspects, including the attempt to remove more brain tissue to find the lesions, leading to an increased risk of neurological damage. In this respect, the use of intraoperative magnetic resonance imaging (IoMRI) can facilitate the localization and margin determination during both intraaxial and extraaxial CCM resections [17, 20]. Moreover, it has been acknowledged that the combined use of IoMRI and awake microsurgical resection can help maximize resection of intraaxial lesions, particularly gliomas [18]. Thus, such an appealing combination might be used for CCM resection. Recently, it has also been suggested that some of the CCM located within eloquent areas, and in patients refusing awake surgical resection, a minimal stereotactic-guided approach can be used [9].

Here, we present a CCM operated under awake conditions, due to location in an eloquent area, and using intraoperative MRI, the former due to patient’s movement upon the awake phase.

## Case presentation

### Surgical considerations

The current gold-standard indication of awake surgery is to preserve neurological functions, including motor, language, and cognitive functions [19], for any type of lesion observed near or within eloquent areas of the brain [15].

**Fig. 1:**
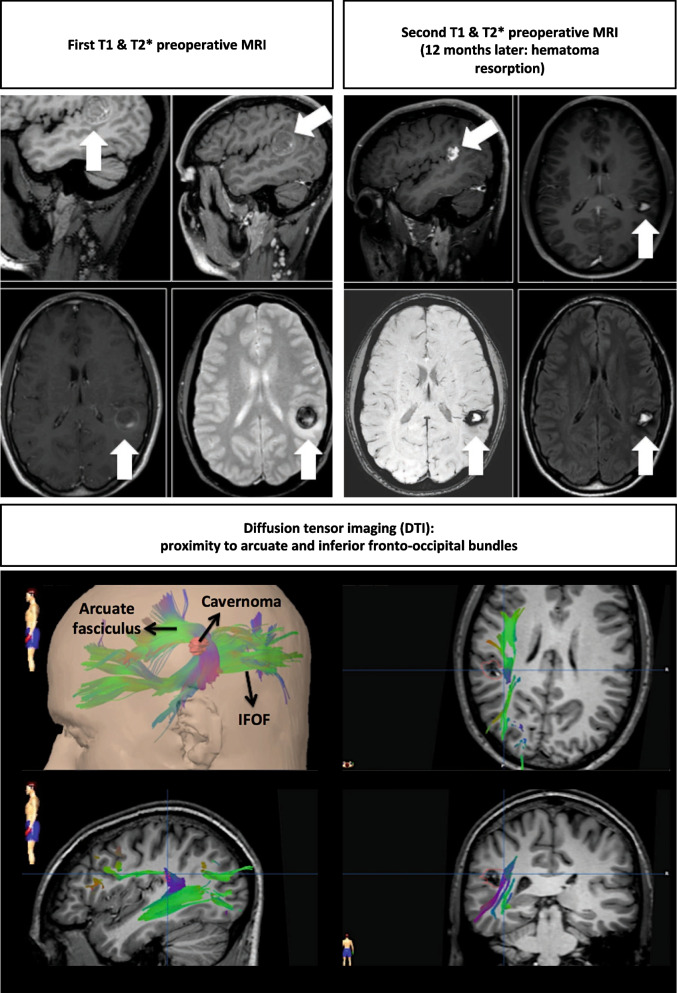
Preoperative MRI related to our patient in the illustrative video

Here, the exact anatomical location was at the interface between the inferior parietal lobule and the posterior temporal lobe (historically described as Wernicke’s area) [2], in the subcortical area of the supramarginal and angular gyri. Moreover, diffusion tensor imaging (DTI, Fig. 1) has shown the CCM at the interface between the arcuate fasciculus (AF) and the inferior fronto-occipital fasciculus (IFOF). The AF belongs to the core perisylvian circuitry underlying language. It represents a subcortical association fiber tract and can be considered as part of the superior longitudinal fasciculus. The tract connects the fronto-opercular cortical sites (i.e., Broca’s motor speech center) with the posterior superior temporal gyrus (i.e., Wernicke’s sensory word area) in the dominant hemisphere, with important individual variations [3]. Disturbance of the AF has been associated with multiple speech disturbances, most classically conduction aphasia [1]. The functional roles of the IFOF are diverse, including in the semantic system, by the connections with the two areas involved in semantics, including the occipital associative extrastriate cortex and the temporo-basal region [10]. It is also considered a middle component, which could play a role in a multimodal sensory-motor integration. Finally, the anterior part is believed to be linked to emotional and behavioral aspects. Recently, the development of DTI has made visualization of white matter association tracts possible *in vivo*, including AF and IFOF [4].

### MRI aspects

DTI is a noninvasive technique that allows subcortical fiber tracts visualization in the healthy brain, but also in the damaged areas [11–14]. DTI sequences were performed either during the presurgical neuronavigation MRI (1.5 T; General Electric; 32 directions; b values, 0 and 1000; slice thickness 4.0; no interslice gap; FOV, 26.0). The mean duration of the DTI sequence was less than 9 minutes. The DTI images, including the color-coded FA maps, were reconstructed using the manufacturer software (DICOM Viewer 3.2.0.410, Philips—Volume Viewer 11.3 Ext. 8, GE) [8].

Before doing an IoMRI evaluation to assess the extent of resection of the cavernoma, the dura and skin were roughly approximated. A sterile field was positioned over the operative one. The head holder we use is the Mayfield MR/X-Ray Skull clamp with Excite 3.0T Adaptator (Integra Lifescience, New Jersey, USA). All patients benefited from an intraoperative 1.5 Tesla MRI (General Electric, Boston, MA). The imaging sequences for neuronavigation were 3D T1 after gadolinium injection with 1-mm slice thickness. Additional sequences, such as 3D FLAIR, diffusion (B1000 and ADC), and T2 with gradient echo, could be performed. An intraoperative tractography was done, as previously stated, due to the fact that the cavernoma was close to functional areas (e.g., Wernicke area). The neuronavigation data update procedure was performed using the automatic co-registration provided by Brainlab Munich, Germany. The quality and accuracy of the co-registration were double-checked by an imaging engineer and the board-certified neurosurgeon [7]. We always discuss the MRI with our neuroradiologist so as to evaluate the extent of resection (EOR).

The surgical microscope (OPMI Pentero Zeiss, Germany) was connected to the imaging network and could be used as neuronavigation.

### Illustrative case (and video of the microsurgical resection)

We illustrate the case of a 27-year-old right-handed Caucasian male, diagnosed with CCM, after presenting with intralesional hemorrhage and epilepsy. His medical history was positive for lymphoblastic leukemia, treated with whole body radiotherapy 8 years before. The DTI has shown the CCM at the interface point between the AF and IFOF. After the hematoma resorption, microsurgical resection is performed under awake conditions and IoMRI setting. Neurological exam was unremarkable.

A left parieto-temporal approach was performed (please see the Additional file 1). We applied the most common approach for awake surgery, meaning the “asleep-awake-asleep” (AAA) technique. By this approach, there is an initial phase of general anesthesia, followed by intraoperative awakening and brain mapping and then finally back to general anesthesia for the end of the surgery [16]. Patient was installed in supine position. A scalp block was performed. The head was fixed in a 3-pin Mayfield, in the intraoperative MRI room. Neuronavigation allowed planning of the future craniotomy, centered on the lesion and the surrounding eloquent cortex.

After incision, scalp and muscle retraction, we performed the skull opening, and further injected the dura and incision margins with local anesthetic (lidocaine 2%, without adrenaline). During the awakening process the patient moved inside the 3-pin Mayfield, thus making neuronavigation unusable.

After complete awakening the patient was calm and cooperative, allowing an imaging update in the intraoperative MRI. The patient was then transferred in the operating room, after co-registration of the MRI with the new head position.

We began the bipolar electrical stimulation mapping with the good cooperation of the patient, who remained fully awake. Thus, we further identified several areas of anomia. These areas were protected, imposing a rectification of the initially planned trajectory for the corticotomy.

A point of entry was chosen where no language disturbance was encountered. We started the microsurgical resection in a perilesional dissection manner. We dissected the CCM and the associated hemosiderin ring from the surrounding brain parenchyma. The microsurgical technique included sharp dissection and piecemeal resection.

Patient remained awake and continued the speech mapping with satisfactory cooperation.

After the resection, the neurosurgeon and neuropsychologist again evaluated the patient and the eloquent areas were considered intact.

No intraoperative focal or generalized seizure was encountered.

**Fig. 2:**
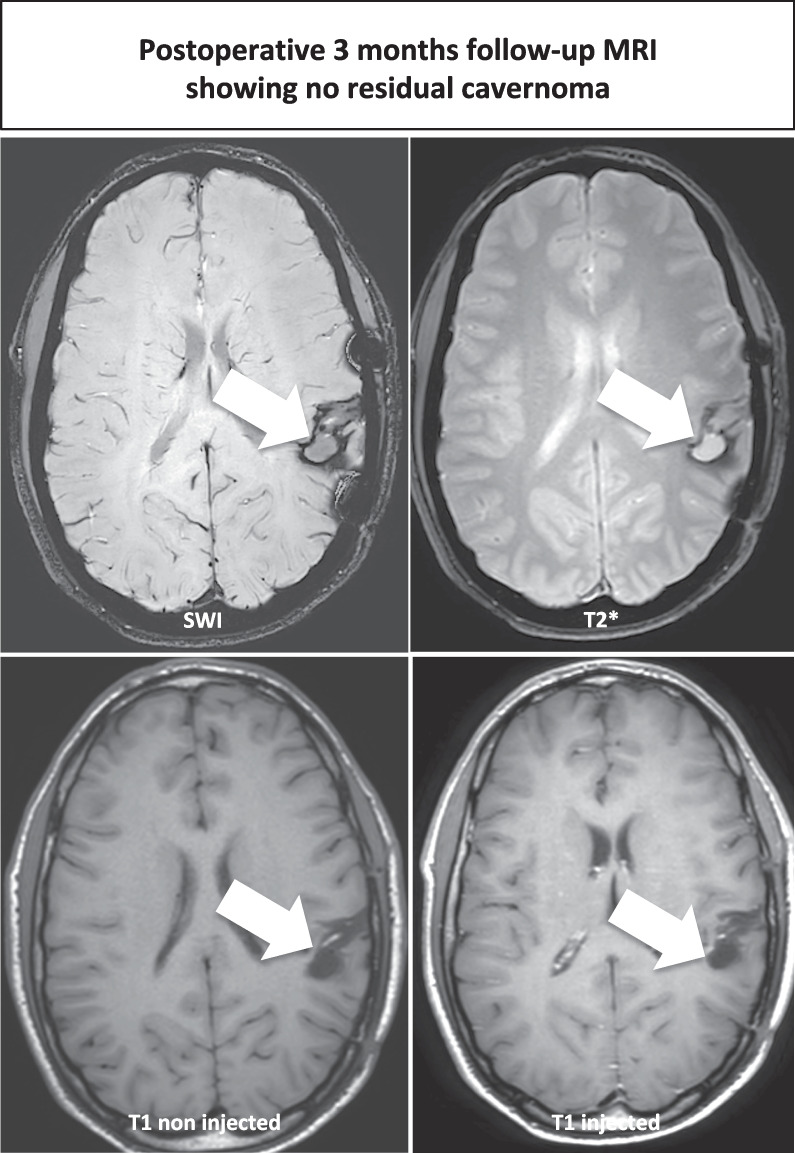
Postoperative MRI assessment. The arrows point out the cavernoma

The 3 months postoperative MRI is presented in Fig. 2, showing complete microsurgical resection. Orthophonic assessment was performed before and after surgery (and was unchanged).

### Limitations

A major aspect, although not a formal limitation, is the higher load of surgical information, which is challenging for surgeons in terms of assimilation. We observed a learning curve for all implicated actors (neurosurgeon, neuropsychologist, anesthesia team), permitting fast reactions in a well-trained team and adjustment of the procedure, as seen in this case. There is a need for multidisciplinary operative team and patient compliance and motivation. Awake surgery should not be offered, in the context of this pathology, to patients with pronounced aphasia, Mini-Mental State Examination less than 23, or with apathic/disorganized behavior.

Of note, several aspects of DTI limitations are of relevance. Various aspects can affect tractography reconstructions: (1) the software used, (2) the technique itself using echo-planar, which may be responsible for geometric distortions, magnetic susceptibility artifacts, and partial volume, and (3) the direct effects of the hematoma on the adjacent white matter, which are mainly of two types: (a) an anisotropy decrease within the hematoma or in the white perilesional substance, due to either hematoma, edema, or destruction of myelin, resulting in a stop or absence of fiber reconstruction, and (b) a displacement of the fibers by the hematoma leading to the reconstruction of aberrant fibers.

## Conclusion

Here, we used combined awake microsurgical resection and IoMRI to completely resect a CCM located within an eloquent area. IoMRI can be a very useful complementary technique included in such cases, particularly when the patient moves during the “awake” phase of the procedure, as such happened in our case. Intraoperative DTI, with the acknowledged limitations, is helpful to evaluate the integrity of the white matter tracts, which are at particular risk in such surgery.

## Supplementary Information

**Additional file 1.** This video presents the case of a 27-year-old right-handed male, diagnosed with CCM, after presenting with intralesional hemorrhage and epilepsy. His medical history was positive for lymphoblastic leukemia treated with whole body radiotherapy 8 years before. The DTI has shown the CCM at the convergence point between the AF and IFOF. After the hematoma resorption, microsurgical resection is performed under awake conditions and IoMRI setting. The surgical trajectory is different as compared with the planned, straightforward one that was initially chosen. The patient moved in the 3-pin Mayfield, making an IoMRI necessary before resection, to upgrade the neuronavigation information. Cortical mapping was performed. The microsurgical resection is complete and performed in piecemeal fashion. Postoperative MRI shows complete resection.

## Data Availability

Data is not available, besides the radiological images, there is no need for further data.
